# Granzyme B secretion by human memory CD4 T cells is less strictly regulated compared to memory CD8 T cells

**DOI:** 10.1186/s12865-014-0036-1

**Published:** 2014-09-24

**Authors:** Lin Lin, Jacob Couturier, Xiaoying Yu, Miguel A Medina, Claudia A Kozinetz, Dorothy E Lewis

**Affiliations:** Division of Infectious Diseases, Department of Internal Medicine, University of Texas Health Science Center at Houston, 6431 Fannin St., MSB 2.112, Houston, TX 77030 USA; Department of HIV/AIDS prevention and control, Shandong Provincial Key Laboratory of Infectious Disease Control and Prevention, Shandong Center for Disease Control and Prevention, Ji Nan, Shandong People’s Republic of China; Academy of Preventive Medicine, Shandong University, Jinan, People’s Republic of China; Department of Pediatrics, Baylor College of Medicine, Houston, TX USA

**Keywords:** ELISA, ELISpot, Flow cytometry, Granzyme B, Memory T cells, Perforin

## Abstract

**Background:**

Granzyme B (GrzB) is a serine proteinase expressed by memory T cells and NK cells. Methods to measure GrzB protein usually involve intracellular (flow cytometry) and extracellular (ELISA and ELISpot) assays. CD8 T cells are the main source of GrzB during immunological reactions, but activated CD4 T cells deploy GrzB as well. Because GrzB is an important mediator of cell death, tissue pathology and disease, clarification of differences of GrzB expression and secretion between CD4 and CD8 T cells is important for understanding effector functions of these cells.

**Results:**

Memory CD4 and memory CD8 T cells were purified from human peripheral blood of healthy donors, and production of GrzB was directly compared between memory CD4 and memory CD8 T cells from the same donors using parallel measurements of flow cytometry (intracellular GrzB), ELISpot (single cell secretion of GrzB), and ELISA (bulk extracellular GrzB). Memory CD8 T cells constitutively stored significantly more GrzB protein (~25%) compared to memory CD4 T cells as determined by flow cytometry (~3%), and this difference remained stable after 24 hrs of activation. However, measurement of extracellular GrzB by ELISA revealed that activated memory CD4 T cells secrete similar amounts of GrzB (~1,000 pg/ml by 1x10^5^ cells/200 μl medium) compared to memory CD8 T cells (~600 pg/ml). Measurement of individual GrzB-secreting cells by ELISpot also indicated that similar numbers of activated memory CD4 (~170/1x10^5^) and memory CD8 (~200/1x10^5^) T cells secreted GrzB. Expression of CD107a further indicated that Grzb is secreted similarly by activated CD4 and CD8 T cells, consistent with the ELISA and ELISpot results. However, memory CD8 T cells expressed and secreted more perforin compared to memory CD4 T cells, suggesting that perforin may be less associated with GrzB function for memory CD4 T cells.

**Conclusions:**

Although measurement of intracellular GrzB by flow cytometry suggests that a larger proportion of CD8 T cells have higher capacity for GrzB production compared to CD4 T cells, ELISpot and ELISA show that similar numbers of activated CD4 and CD8 T cells secrete similar amounts of GrzB. Secretion of GrzB by activated CD8 T cells may be more tightly controlled compared to CD4 T cells.

## Background

Granzyme B (GrzB) is a serine proteinase important for its role in mediating cellular apoptosis as well as acting as an extracellular protease. GrzB is expressed primarily by activated memory CD8 and memory CD4 T cells, and NK and NKT cells during infections and inflammation. Other leukocytes such as dendritic cells, macrophages, B cells, and mast cells can express GrzB but such expression is more limited [1-5]. GrzB is upregulated in CD8 T cells after CD3/TCR activation, as well as by common γ-chain cytokines including IL2 and IL15. In memory and effector CD4 T cells, Treg, Th1, and Th17 cells, GrzB is also induced after TCR activation and similar cytokines, as well as by TLR ligands [6,7]. Similarly to memory CD8 T cells, memory CD4 T cells also kill virally-infected or tumor cells via GrzB [8-10]. GrzB expression and bioactivity appears to be comparable amongst CD4 and CD8 T cells, but no studies have directly compared GrzB production between human CD4 and CD8 T cells. Differences in GrzB expression, storage, and secretion suggest that GrzB functions may differ between CD4 and CD8 T cells in immunity and disease.

Studies examining expression and functional activity of GrzB or GrzB-associated molecules such as perforin or CD107a (LAMP-1) in CD4 and CD8 T cells utilize mainly western blot, flow cytometry, and CTL killing assays. For example, previous comparison of GrzB expression in human CD4 and CD8 T cells by flow cytometry showed that CD8 T cells express more intracellular GrzB protein, however, comparison of extracellular GrzB between CD4 and CD8 T cells was not examined [11]. Our previous work directly compared human memory CD4 and memory CD8 T cells by flow cytometry and we found that resting and activated memory CD4 T cells store little to no GrzB protein intracellularly, whereas resting and activated memory CD8 T cells store substantially more GrzB [12]. However, ELISA showed that activated memory CD4 and memory CD8 T cells secreted similar amounts of GrzB. In another study, using immortalized human HSV- and EBV-specific CD4 CTL clones, CD8 CTL’s were shown to express significantly more perforin mRNA compared to CD4 CTL’s, and target cell killing was similar between CD4 and CD8 CTL’s (although GrzB was not examined) [13]. In a mouse model of LCMV infection, direct comparison of antigen-specific CD4 and CD8 CTL’s by flow cytometry showed that CD8 T cells express more GrzB and CD107a. However, in vivo CTL killing measurements showed that CD4 T cells eliminate target cells with comparable efficiency and magnitude as CD8 T cells [14,15]. Thus, CD4 and CD8 T cells may differ in GrzB synthesis, storage and secretion, but clarification of discrepancies between methods to measure intracellular and extracellular GrzB is necessary to better understand the roles of GrzB in effector functions and tissue pathology mediated by CD4 T cells.

The goal of the present study was to directly compare GrzB production by human memory CD4 and memory CD8 T cells (ie. purified from the same donor and studied in parallel) with the common assays of flow cytometry, ELISpot, and ELISA. Each of these gives different information about the effector function of a T cell. Flow cytometry indicates the intracellular content of single cells, and ELISpot measures how much is secreted from individual cells and is thus conceptually similar to flow cytometry measurement. Lastly, ELISA indicates how much GrzB is secreted globally, but does not indicate how many cells the material came from. Our studies seek to clarify differences of intracellular GrzB expression vs. actual secretion by memory T cells. Differences in GrzB protein storage and secretion were observed between resting and activated memory CD4 and memory CD8 T cells. Activated memory CD4 T cells usually secreted similar amounts of GrzB compared to memory CD8 T cells (ELISpot and ELISA), despite memory CD8 T cells storing significantly more GrzB as indicated by flow cytometry. Thus, activated memory CD4 T cells are major sources of extracellular GrzB, and the use of flow cytometry or western blot alone to measure intracellular GrzB does not accurately predict secretion levels.

## Methods

### Cells and culture

Human memory CD4+CD45RO+ and memory CD8+CD45RO+ T cells were purified from PBMC of healthy buffy coat donors. PBMC were first isolated from buffy coats (Gulf Coast Regional Blood Center, Houston, TX) by using Ficoll-Paque PLUS (GE Healthcare). Memory CD4 and memory CD8 T cells were then negatively selected from PBMC with magnetic bead-based EasySep kits (Stemcell Technologies). Purities of memory CD4+CD45RO+ T cells were usually >90% and memory CD8+CD45RO+ T cells >80% as determined by flow cytometry. Natural killer cells were purified from PBMC with negative selection kits (Stemcell Technologies), and CD3-CD56+ purity was >90%. Cells were cultured at 37°C + 5%CO_2_ in complete RPMI-1640 medium, supplemented with 10% FBS, 0.1 mM MEM nonessential amino acids, 2mM sodium pyruvate, 2mM HEPES, 1× antibiotic-antimycotic and 2mM L-glutamine.

Most experiments involved no stimulation (UT) or stimulation of 1-5×10^5^ memory CD4 and memory CD8 T cells, or NK cells (200 μl/well of 96-well flat-bottom plates, or 1 ml/well of 24-well plates, as indicated) for 24 hrs with 1 ml/ml coated CD3 (clone UCHT1, BD Biosciences) or soluble CD3 (clone CD3-2, Mabtech) + 1 μg/ml CD28 (clone CD28.2, BD Biosciences) mabs. For some experiments, cells were stimulated with 100 ng/ml recombinant IL2 (Biolegend) or soluble CD3 mab alone for 24 hrs with or without brefeldin (GolgiPlug, BD Biosciences).

### Flow cytometry

At appropriate time points, memory CD4 and memory CD8 T cells were examined for activation (surface and intracellular CD69) and intracellular GrzB expression by flow cytometry. Cells were washed with PBS/2%FBS and incubated with CD69-APC or -PE mabs (Biolegend and R&D Systems) for 30 mins at 4°C. Cells were then washed and fixed and permeabilized with Cytofix/Cytoperm solution (BD Biosciences) for 30 mins at 4°C. After fixation, cells were washed with Perm/Wash solution (BD Biosciences), then incubated with GrzB-AlexaFluor-700, -PE, or –APC mabs (BD Biosciences and Invitrogen) for 30 mins at 4°C. Cells were washed and analyzed with flow cytometer. Cells were also stained with appropriate isotype controls to assess nonspecific binding. Data was acquired with Gallios Flow Cytometer and analyzed with Kaluza1.2 software (Beckman-Coulter). For measurement of CD107a coexpression with GrzB, CD107a-FITC (BD Biosciences) was added to cells at the start of the experiment. Cells were then cultured with brefeldin for 3-4 hrs. Cells were then harvested and stained for GrzB as described above. For measurement of intracellular perforin expression with GrzB, cells (memory T cells and NK cells) were fixed/permeabilized with Cytofix/Cytoperm solutions, and stained with perforin-PE (eBioscience) and GrzB-APC mabs. For measurement of intracellular cytokine expression with GrzB, memory T cells were fixed/permeabilized with Cytofix/Cytoperm solutions, and stained with GrzB-PE and either IFNγ-FITC, IL4-FITC, or IL17A-Pacific blue mabs (Biolegend and eBioscience). For measurement of GrzB expression by Tregs, memory CD4 T cells were stained using Foxp3 fixation/permeabilization solutions (eBiocience) and CD25-V450 (BD Biosciences), Foxp3-PE (eBioscience), and GrzB-APC.

### ELISpot

GrzB ELISpot’s were performed in accordance with manufacturer instructions (Mabtech). 1×10^5^ memory CD4 and memory CD8 T cells were cultured (200 μl/well) for 24 hrs in 96-well flat-bottomed nitrocellulose plates (MultiScreen-HTS, Millipore). Wells were first prepared by treating with 70% ethanol for 2 mins and rinsing with sterile H_2_0. Wells were then coated overnight at 4°C with 15 μg/ml GrzB mab (clone GB10). After GB10 coating, wells were washed with PBS, and pre-incubated with cell culture RPMI-1640 medium (200 μl/well) for 30 mins at room temp. Cells were then added to appropriate wells and either unstimulated (UT) or costimulated with 1 μg/ml soluble CD3 (clone CD3-2, Mabtech) + soluble CD28 mabs (clone CD28.2, BD Biosciences) for 48 hrs wrapped in aluminum foil at 37°C + 5%CO_2_. After the treatment period, cells were gently harvested for flow cytometry staining, and wells were washed with PBS. 1 μg/ml biotinylated GrzB detection mabs (clone GB11) were added for 2 hrs at room temp. Wells were washed and streptavidin-HRP added for 1 hr. Wells were washed and TMB substrate added until spot development (10-30 mins). Color development was stopped with H_2_0, and plates were air-dried, wrapped, and stored at room temp. until spot analysis. Spots were analyzed using CTL-ImmunoSpot S4 analyzer (Cellular Technology Limited).

### ELISA

At appropriate time points, culture supernatants were harvested, spun free of cells, and frozen at -20°C for extracellular GrzB or perforin measurements by sandwich ELISA kit (Mabtech). GrzB ELISA was performed according to manufacturer instructions. MaxiSorp flat-bottom 96-well plates (Nunc) were first coated with 2 μg/ml GrzB mab (clone GB10) overnight at 4°C. Wells were then washed 2x with PBS, then blocked for 1 hr at room temperature with PBS/0.05%Tween-20/0.1%BSA. Samples were added for 2 hrs at room temp., wells washed, then 1 μg/ml GrzB-biotin (clone GB11) added for 1 hr. Wells were then washed, and 1 μg/ml streptavidin-HRP added for 1 hr. After streptavidin-HRP incubation, wells were washed and TMB substrate added for up to 20 mins (Biolegend). Color development was stopped with TMB stop solution and OD (450 nm absorbance) measured with plate reader (Tecan Sunrise). Sample concentrations were determined with standard curve (range of 23-2,000 pg/ml) after blank subtraction.

For measurement of extracellular perforin, an ELISA kit was used in accordance with manufacturer instructions (Mabtech). Samples were added to perforin mab-precoated plates for 2 hrs. Wells were then washed and detection mab added for 60 mins. Wells were washed and Streptavidin-HRP added for 60 mins. Wells were washed and TMB substrate added for 15 mins, followed by addition of stop solution. OD was measured at 450 nm absorbance and concentrations determined with standard curve (range of 36-10,000 pg/ml).

### Statistics

The values were presented by mean and standard error. Wilcoxon signed-rank tests were used to compare the difference between untreated and costimulated conditions, and between CD4 and CD8 cells for each assay, respectively. The Spearman correlation coefficients were used to assess the correlation among continuous variables. A p-value <0.05 was considered statistically significant. SAS software version 9.3 (SAS Institute, Cary, NC) was used for analyses.

## Results and discussion

### Differential storage and secretion of granzyme B between memory CD4 and memory CD8 T cells

Both memory CD4 and CD8 T cells express GrzB and utilize GrzB for CTL functions. The use of flow cytometry for measurement of GrzB, perforin, or CD107a suggests that CD8 T cells produce more GrzB and may be more potent CTL effectors than CD4 T cells [11-13]. However, murine CTL lysis assays show that CD4 and CD8 T cells kill target cells with similar efficacy, indicating lack of correlation between intracellular GrzB expression and extracellular GrzB secretion by CD4 and CD8 T cells [14]. To further clarify these differences, we directly compared resting or activated (24 hrs CD3/CD28 costimulation) 1×10^5^ (cultured in 200 μl medium) purified human memory CD4+CD45RO+ and CD8+CD45RO+ T cells from the same donor using in parallel multiparameter flow cytometry, ELISpot, and ELISA. Multiparameter flow cytometry measures intracellular GrzB protein which can be examined in conjunction with other cellular markers, ELISpot measures individual GrzB-secreting cells, and ELISA measures total amounts of GrzB released by cells in a specified volume.

**Figure 1:**
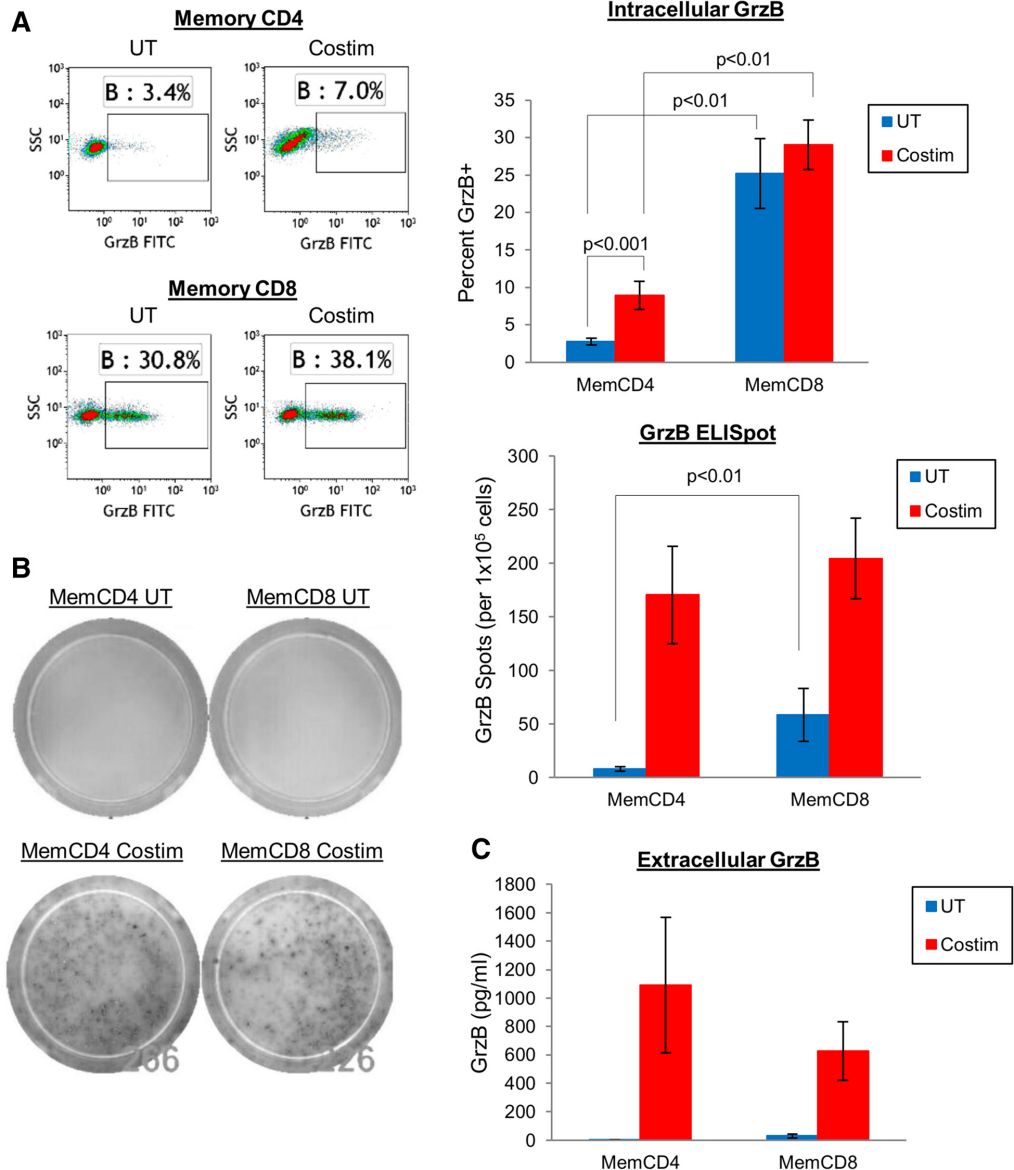
**Comparison of intracellular and extracellular GrzB protein levels by memory CD4 and memory CD8 T cells. (A)** Representative flow cytometry dotplots and mean ± sem intracellular GrzB protein expression by 1×10^5^ untreated (UT) or activated (CD3/CD28 costimulation) memory T cells after 24 hrs culture (n = 10-13). **(B)** Representative ELISpot wells and mean ± sem GrzB spots (per 1×10^5^ cells) of untreated or activated (CD3/CD28 costimulation) memory T cells after 24 hrs culture (n = 10-13). **(C)** Extracellular GrzB production (ELISA) by 1×10^5^ untreated or activated (CD3/CD28 costimulation) memory T cells after 24 hrs culture (mean ± sem, n = 10-13).

Measurement of intracellular GrzB by flow cytometry showed that resting and activated memory CD8 T cells stored significantly more GrzB compared to memory CD4 T cells (Figure 1A). Resting memory CD8 T cells expressed 25.2 ± 4.7% intracellular GrzB protein, which did not significantly increase after 24 hrs costimulation to 29.0 ± 3.3% (p = 0.38, n = 10). Resting memory CD4 T cells expressed little to no intracellular GrzB (2.8 ± 0.5%), but GrzB expression was significantly increased by costimulation to 8.9 ± 1.9% (p = 0.0002, n = 13). Additionally, resting and activated memory CD8 T cells expressed significantly more intracellular GrzB compared to resting and activated memory CD4 T cells (p = 0.002). These data corroborate previous reports of intracellular GrzB protein measurements by flow cytometry or western blot showing higher expression by CD8 compared to CD4 T cells [11-15]. However, unlike memory CD8 T cells, GrzB synthesis is actually upregulated by memory CD4 T cells during T cell activation. These data suggest that activated memory CD8 T cells secrete pre-synthesized GrzB (stored in lysosomes) directly from granules, whereas activated memory CD4 T cells secrete pre-stored and newly synthesized GrzB. This GrzB synthesis step by memory CD4 T cells may also be consistent with a previously reported observation of a less rapid, but ultimately comparable, CTL activity (target cell lysis in a 6 hrs assay) by antigen-specific CD4 T cells compared to CD8 T cells in a murine model of LCMV infection [14].

Although the use of ELISpot to examine GrzB release by CD8 T cells is well-described [16-20], less clear is how well this method measures GrzB release by memory CD4 T cells. By contrast to flow cytometry data, measurement of secreted GrzB with ELISpot showed that the number of GrzB-secreting cells was comparable between activated memory CD4 and memory CD8 T cells (Figure 1B). ELISpot assays showed that resting memory CD4 T cells did not secrete GrzB after 24 hrs culture. However, some resting memory CD8 T cells constitutively secreted GrzB (~60 GrzB spots per 1×10^5^ resting memory CD8 T cells), consistent with more storage of GrzB and low levels of stimulation in vitro. After 24 hrs costimulation, similar numbers of GrzB spots were detected by activated memory CD4 (171 ± 46 GrzB spots per 1×10^5^ memory CD4 T cells, n = 13) and memory CD8 T cells (205 ± 38 GrzB spots per 1×10^5^ memory CD8 T cells, n = 10, p = 0.11). Although ELISpot sensitivity may more be limited beyond a particular range of spot formation, these data suggest that similar numbers of activated memory CD4 and memory CD8 T cells secreted GrzB.

Consistent with the ELISpot assays, measurement of bulk supernatant GrzB by ELISA showed that activated memory CD4 and memory CD8 T cells secrete similar amounts of GrzB (Figure 1C). Extracellular GrzB was not detected by resting memory CD4 T cells after 24 hrs culture, but ~30 pg/ml GrzB was detected by resting memory CD8 T cells. After 24 hrs costimulation, extracellular GrzB was 1,093 ± 476 pg/ml and 627 ± 206 pg/ml for activated memory CD4 and memory CD8 T cells, respectively. In conjunction with the ELISpot data, these ELISA data indicate that at a single cell level, similar amounts of GrzB are secreted per activated memory CD4 (1,093 pg/ml GrzB per 171 cells) and memory CD8 (627 pg/ml GrzB per 205 cells) T cell. These extracellular GrzB data may also suggest that much of the pre-stored intracellular GrzB in memory CD8 T cells indicated by flow cytometry may not actually be secreted. These similarities of extracellular GrzB production between memory CD4 and CD8 T cells are consistent with previous reports of GrzB effector function demonstrating comparable CTL activity by CD4 and CD8 T cells using in vitro-generated virus-specific human CTL clones [13], and in vivo LCMV-specific CTL assays with mice [14]. Thus, the simultaneous use of these intracellular and extracellular GrzB measurements confirm that resting memory CD8 T cells store more GrzB compared to memory CD4 T cells. However, similar numbers of activated memory CD4 T cells secrete similar amounts of GrzB as memory CD8 T cells, which are likely achieved by higher levels of de novo GrzB synthesis.

### Memory T cell granzyme B assay correlations, and lack of correlation of GrzB production between memory CD4 and memory CD8 T cells within single donors

**Figure 2:**
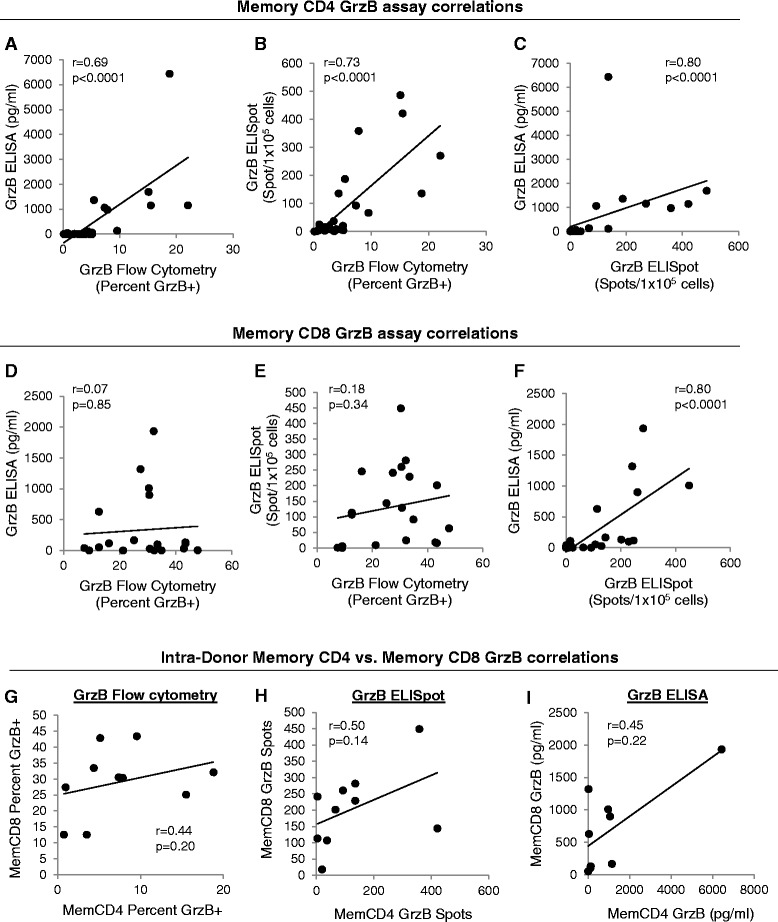
**Inter-assay correlations between intracellular and extracellular GrzB assays of memory T cells, and intra-donor correlations of GrzB production between memory T cells. (A-F)** Spearman correlations between intracellular (flow cytometry) and extracellular (ELISA and ELISpot) GrzB measurements of 1×10^5^ untreated (UT) or activated (CD3/CD28 costimulation) memory CD4 **(A-C)** or memory CD8 **(D-F)** T cells (n = 20-26). **(G-I)** Spearman correlations comparing GrzB production (costimulation values) of memory CD4 vs. memory CD8 T cells within single donors as measured by flow cytometry, ELISpot, or ELISA (n = 9-10).

We examined correlations between each GrzB assay for memory CD4 or memory CD8 T cells (Figure 2A-F). Inter-assay correlations of GrzB measurement between flow cytometry, ELISpot, and ELISA suggested that intracellular GrzB measured by flow cytometry correlates with extracellular GrzB as measured by ELISpot and ELISA for memory CD4 T cells, but not for memory CD8 T cells. For memory CD4 T cells, the Spearman correlation coefficient between GrzB flow cytometry and ELISA was r = 0.69 (p < 0.0001, n = 26), and r = 0.77 (p < 0.0001) between GrzB flow cytometry and ELISpot (Figure 2A-B). However, because memory CD8 T cells store more intracellular GrzB compared to memory CD4 T cells without proportionally secreting higher amounts of GrzB, no correlation was observed between GrzB flow cytometry and ELISA (r = 0.044, p = 0.85, n = 20), or between GrzB flow cytometry and ELISpot (r = 0.23, p = 0.34) (Figure 2D-E). Additionally, these weak correlations between intracellular and extracellular GrzB of memory CD8 T cells are unlike those for cytokines such as IFNγ or IL2 which strongly correlate between intracellular and extracellular levels for CD4 and CD8 T cells. For example, human resting T cells do not express or pre-store intracellular IFNγ or IL2 (measured by flow cytometry), but upregulate these cytokines intracellularly and extracellularly (measured by ELISpot or ELISA) upon T cell activation, and cytokine detection between flow cytometry and either ELISpot or ELISA significantly correlate (r = 0.6-0.9) [21-26]. Since memory CD4 T cells do not pre-store GrzB at levels similar to memory CD8 T cells, these inter-assay correlations between intracellular and extracellular GrzB of memory CD4 T cells resemble the correlations of other secreted factors that are not pre-stored, such as cytokines. Lastly, the correlation between GrzB ELISpot and ELISA was comparable for memory CD4 (r = 0.80, p < 0.0001, Figure 2C) and memory CD8 (r = 0.80, p < 0.0001, Figure 2F) T cells. Thus, the correlation between intracellular and extracellular GrzB is stronger for memory CD4 T cells compared to memory CD8 T cells.

The bulk data in Figure 1 suggests that intracellular GrzB is increased by activated memory CD4 but not by memory CD8 T cells (Figure 1A), whereas extracellular GrzB is increased by both activated memory CD4 and memory CD8 T cells (Figure 1B-C). However, when we examined GrzB production by memory CD4 and memory CD8 T cells within individual donors to determine if increased GrzB production by memory CD4 T cells is also associated with increased GrzB production by memory CD8 T cells, and vice versa (ie. if GrzB production capacity is inherent to a person or to memory T cell subsets), correlations of GrzB production between memory CD4 and memory CD8 T cells from the same donor were weak (r < 0.50, Figure 2G-I). Spearman correlations of intracellular and extracellular GrzB production (costimulation values) also indicated that no significant correlations exist between memory CD4 and memory CD8 T cells for any assay (p = 0.14-0.22). The use of GrzB absolute or percent changes (change from UT to costimulation) also yielded no significant correlations between memory CD4 and memory CD8 T cells for each assay (data not shown). Although Figure 1 shows that GrzB production by memory T cells is generally increased by T cell activation, these data in Figure 2 suggest that within an individual, the induction and secretion levels of GrzB are different and unique between memory CD4 and memory CD8 T cells.

### Association of CD107a degranulation marker with extracellular, but not intracellular, granzyme B of memory T cells

**Figure 3:**
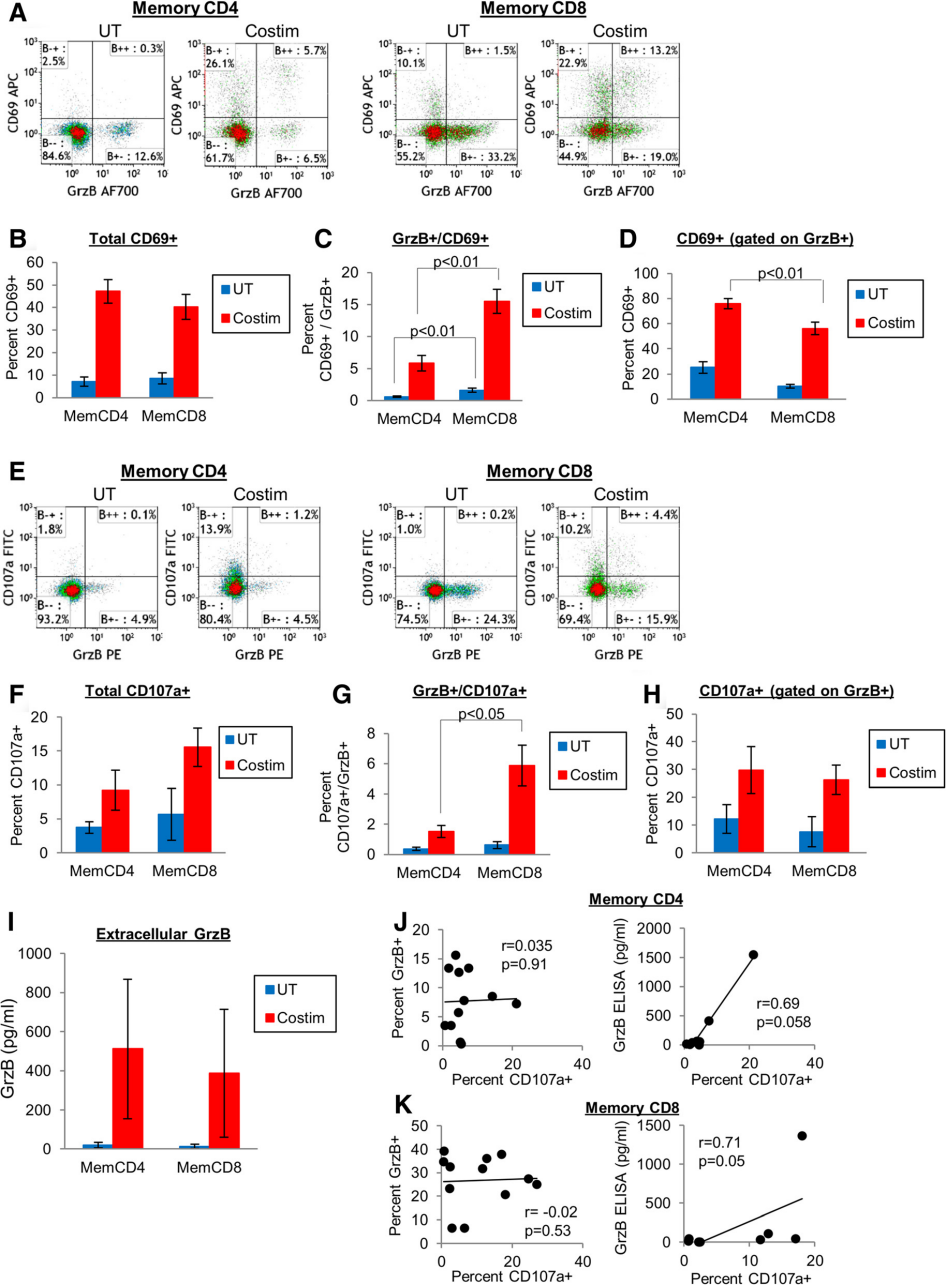
**Association of memory T cell GrzB secretion with activation and degranulation markers. (A-D)** Memory T cell flow cytometry dotplots and mean ± sem (n = 16-17) total surface CD69 expression or CD69+/GrzB+ coexpression (gated on total memory CD4 or memory CD8 T cells), or CD69 expression gated on GrzB+ memory CD4 or memory CD8 T cells. **(E-H)** Flow cytometry dotplots and mean ± sem (n = 6) total surface CD107a expression or CD107a+/GrzB+ coexpression (gated on total memory CD4 or memory CD8 T cells), or CD107a expression gated on GrzB+ memory CD4 or memory CD8 T cells. **(I)** GrzB ELISA of memory T cells conducted in parallel with CD107a experiments. **(J-K)** Spearman correlations between CD107a and intracellular (flow cytometry) or extracellular (ELISA) GrzB of memory T cells (n = 8-12).

We examined the association of memory T cell GrzB with activation and degranulation markers, which more accurately indicates GrzB-expressing cells that are also actively secreting GrzB. CD69 is an early T cell activation marker that is transiently upregulated by activated T cells, and CD107a (LAMP-1) is a lysosomal protein detectable by flow cytometry surface staining after transient lysosomal fusion to the cellular membrane and release of granule proteins during T cell activation. Surface expression of CD107a is also associated with perforin release by human CD8 T cells, and correlates with CTL effector function [18,27]. After 24 hrs costimulation, total CD69 expression by memory CD4 (~47%) and memory CD8 (~40%) T cells were similar (Figure 3B). If gated on total memory CD4 or memory CD8 T cells, CD69 coexpression with GrzB (CD69+/GrzB+) was higher by activated memory CD8 (15.5 ± 1.9%) compared to memory CD4 (5.9 ± 1.2%, p = 0.001, n = 16-17) T cells (Figure 3C). However, out of the GrzB+ memory CD4 or memory CD8 T cells, CD69 expression was higher by memory CD4 (75.8 ± 4.1%) compared to memory CD8 T cells (56.2 ± 4.9%, p < 0.01, Figure 3D), indicating that more GrzB-expressing memory CD4 T cells are also activated compared to memory CD8 T cells.

After 24 hrs costimulation, total CD107a expression was 9.2 ± 2.9% in activated memory CD4, and 15.6 ± 2.9% in memory CD8 T cells (p = 0.15, n = 6, Figure 3F). If gated on total memory CD4 or memory CD8 T cells, CD107a+/GrzB+ coexpression was 1.5 ± 0.4% by memory CD4, but higher at 5.9 ± 1.3% by memory CD8 T cells (p = 0.03, Figure 3G), which may more accurately corroborate the frequency of less than 1% activated memory T cells that secrete GrzB as indicated by ELISpot in Figure 1B. But unlike the difference of CD69 expression (out of GrzB-expressing memory T cells) between activated memory T cells shown in Figure 3D, CD107a expression by activated GrzB+ memory T cells was similar (Figure 3H). Additionally, the parallel extracellular GrzB levels (ELISA) for these experiments were similar between activated memory T cells (Figure 3I), suggesting that CD107a is more accurate than CD69 as an indicator of GrzB secretion by activated memory T cells. For memory CD4 T cells, the Spearman correlation of CD107a with intracellular GrzB was r = 0.035 (p = 0.91, n = 12), and r = 0.69 (p = 0.058, n = 8) with extracellular GrzB (Figure 3J). Similarly for memory CD8 T cells, the correlation of CD107a with intracellular GrzB was r = -0.02 (p = 0.53), and r = 0.71 (p = 0.05) with extracellular GrzB (Figure 3K). Despite increased surface expression of CD107a and release of GrzB by activated memory CD8 T cells (compared to UT), intracellular GrzB of memory CD8 T cells does not significantly change after activation as shown by flow cytometry in Figure 1A, which may be consistent with an earlier report that demonstrates by flow cytometry that increased surface expression of CD107a and CD107b by human CD8 T cells is associated with loss of intracellular perforin [27]. For memory CD4 T cells, both CD107a and intracellular GrzB are associated with GrzB secretion to similar degrees, and increased surface expression of CD69 and CD107a by activated memory CD4 T cells is further associated with the increased intracellular GrzB expression shown by flow cytometry in Figure 1A. These data further corroborate Figure 1, in which activated memory CD8 T cells secrete GrzB mainly from pre-stored granules, whereas GrzB secretion by memory CD4 T cells is likely derived from pre-stored and newly synthesized GrzB to achieve the similar extracellular GrzB levels. Despite higher intracellular storage of GrzB by activated memory CD8 compared to memory CD4 T cells (as shown by flow cytometry in Figure 1A), CD107a expression by activated GrzB+ memory T cells, as well as the number of GrzB-secreting cells (as indicated by ELISpot in Figure 1B), were similar. This may suggest that a higher proportion of activated GrzB+ memory CD4 T cells secrete and deplete their GrzB stores. This contrasts with higher retention of intracellular GrzB by activated memory CD8 T cells, since ELISpot numbers would be substantially higher if all activated CD8 T cells released their intracellular GrzB stores. These differences may allow memory CD8 T cells to be more sustained serial killers of target cells, whereas memory CD4 T cells may express GrzB for shorter-term functions [28-30].

### Less association of granzyme B with perforin by memory CD4 T cells compared to memory CD8 and natural killer cells

We next examined GrzB production by memory T cells in conjunction with perforin, and also in comparison with natural killer (NK) cells, abundant producers of GrzB and perforin. In addition to GrzB, CD4 T cells express perforin to eliminate target cells [8,31,32]. Memory CD4+CD45RO+ and memory CD8+CD45RO+ T cells, and CD56+ NK cells, were purified from peripheral blood of the same donor and cultured in parallel, with or without the intracellular protein transport inhibitor brefeldin. Memory T cells and NK cells (5×10^5^ cells in 1 ml medium) were stimulated for 24 hrs with IL2 (100 ng/ml). Additionally, memory T cells were alternatively stimulated with soluble anti-CD3 mabs only (1 μg/ml), as CD28 expression by memory CD8 T cells (~80%) was modestly lower compared to memory CD4 T cells (~95%, data not shown), which may result in memory CD4 T cells receiving stronger stimulation by CD3/CD28 costimulation compared to memory CD8 T cells. Agonism of other costimulatory receptors such as ICOS or TNF superfamily members may further yield different results. Intracellular and extracellular perforin and GrzB were measured by flow cytometry and ELISA.

**Figure 4:**
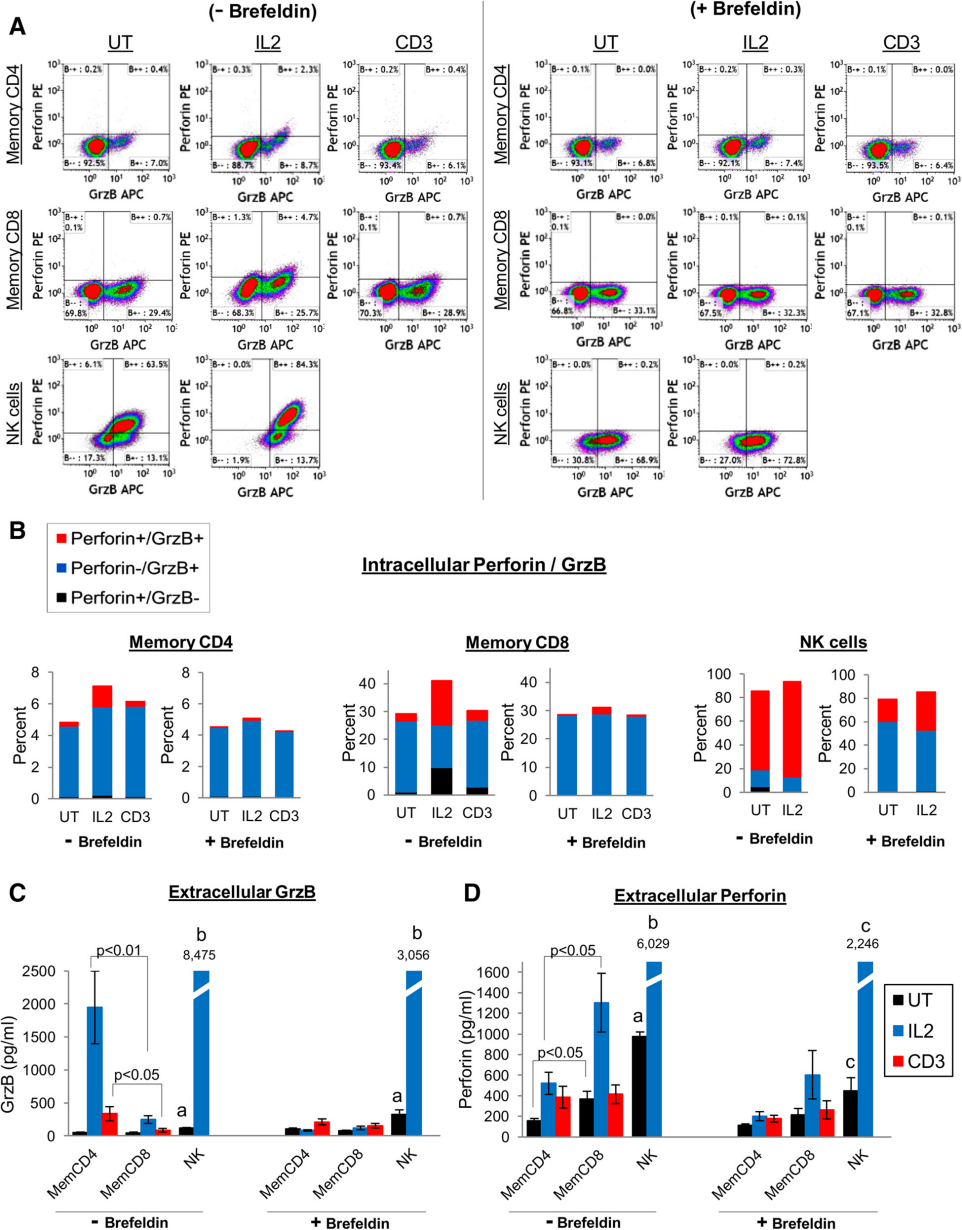
**Comparison of GrzB and perforin expression between memory CD4, memory CD8, and natural killer cells. (A)** Representative GrzB/perforin dotplots of memory CD4, memory CD8, and NK cells. Cells were purified from peripheral blood, and 5×10^5^ cells (in 1 ml medium) were untreated (UT) or stimulated with IL2 (100 ng/ml) or anti-CD3 only mabs (1 μg/ml) for 24 hrs +/- brefeldin. **(B)** Mean (n = 4) distribution of perforin/GrzB subsets of memory CD4, memory CD8, and NK cells. **(C-D)** Extracellular GrzB and perforin production by memory CD4, memory CD8, and NK cells after 24 hrs culture +/- brefeldin (mean ± sem, n = 3-4, ^a^p < 0.05 comparing UT NK cells to UT memory CD4 and memory CD8 T cells, ^b^p < 0.05 comparing IL2-treated NK cells to IL2-treated memory CD4 and memory CD8 T cells, ^c^p < 0.05 comparing UT or IL2-treated NK cells to UT or IL2-treated memory CD4 T cells).

Figure 4A-B shows representative perforin/GrzB flow cytometry dotplots (Figure 4A) and the mean distribution of intracellular perforin/GrzB subpopulations (Figure 4B) for memory T cells and NK cells (n = 4). For memory CD4 T cells, only perforin-/GrzB+ cells were mainly observed in all conditions (~4-6%, with and without brefeldin), and a small percentage of perforin+/GrzB+ cells were observed by IL2 stimulation (1.3 ± 0.6%, without brefeldin). However, memory CD8 T cells expressed more intracellular perforin and GrzB. In the absence of brefeldin, UT memory CD8 T cells were 1.1 ± 0.9% perforin+/GrzB-, 25.5 ± 1.5% perforin-/GrzB+, and 2.6 ± 1.3% perforin+/GrzB+. IL2 stimulation changed these perforin/GrzB distributions by increasing perforin expression (10.0 ± 6.5% perforin+/GrzB-, and 15.9 ± 7.3% perforin+/GrzB+), whereas CD3 stimulation did not affect the perforin/GrzB expression patterns. In the presence of brefeldin, perforin expression was mitigated and memory CD8 T cells were mostly perforin-/GrzB+ (~28% for all conditions). As expected, intracellular perforin and GrzB expression was substantially higher for NK cells compared to memory T cells. In the absence of brefeldin, NK cells were mostly perforin+/GrzB+ (UT NK cells were 14.1 ± 4.0% perforin-/GrzB+ and 66.1 ± 7.3% perforin+/GrzB+, and IL2-stimulated cells were 11.9 ± 3.2% perforin-/GrzB+ and 80.7 ± 6.1% perforin+/GrzB+), by contrast to memory T cells which were mostly perforin-/GrzB+. But in the presence of brefeldin, this distribution changed to mostly perforin-/GrzB+ (59.3 ± 12.1% perforin-/GrzB+ and 18.9 ± 10.5% perforin+/GrzB+ by UT cells, and 51.6 ± 17.5% perforin-/GrzB+ and 32.5 ± 18.4% perforin+/GrzB+ by IL2-stimulated cells), which was similar to the perforin mitigation by brefeldin of memory CD8 T cells. Thus, intracellular perforin is marginally expressed with GrzB by stimulated memory CD4 T cells, whereas perforin is more associated with GrzB in memory CD8 T cells and NK cells.

Despite little to no intracellular perforin expression by memory CD4 T cells, perforin was secreted with GrzB by stimulated memory CD4 T cells as determined by ELISA, but in a pattern distinct from memory CD8 T cells (Figure 4C-D). In the absence of brefeldin, IL2- and CD3-stimulated memory CD4 T cells released 1,950 ± 549 pg/ml and 336 ± 108 pg/ml GrzB, respectively, more than memory CD8 T cells which released 249 ± 58 pg/ml and 86 ± 26 pg/ml GrzB (p < 0.05). However, UT and IL2-stimulated memory CD8 T cells released 372 ± 71 pg/ml and 1,304 ± 284 pg/ml perforin, which was more than memory CD4 T cells which released 159 ± 20 pg/ml and 521 ± 108 pg/ml perforin (p < 0.05). Perforin release by CD3-stimulated memory T cells was similar (385 ± 106 pg/ml by memory CD4, and 415 ± 89 pg/ml by memory CD8 T cells). As expected, UT and IL2-stimulated NK cells secreted substantially more perforin (977 pg/ml by UT and 6,029 pg/ml by IL2-stimulated cells) and GrzB (119 pg/ml by UT and 8,475 pg/ml by IL2-stimulated cells) compared to memory T cells in the absence of brefeldin (p < 0.05). In the presence of brefeldin, perforin and GrzB release by stimulated memory T cells was reduced nearly to baseline (UT) levels, and reduced ~2-3 fold by NK cells. An unclear effect of brefeldin upon resting memory T cells and NK cells was observed, in which a modest decrease of constitutive perforin release, but an increase of GrzB release, by UT memory T cells and NK cells occurred. It is possible that intracellular storage of GrzB may be more disassociated with perforin by resting T cells and NK cells compared to activated states. Additionally, IL2-stimulated memory CD4 T cells and NK cells appear to secrete more GrzB than perforin, whereas memory CD8 T cells secrete more perforin than GrzB. Lastly, despite the release of perforin and GrzB by stimulated memory T cells and NK cells (as shown by ELISA), intracellular accumulation (or increased intracellular expression) of perforin and GrzB in stimulated cells did not occur in the presence of brefeldin (compared to stimulated cells in the absence of brefeldin). This is likely due to glycosylation requirements of perforin and GrzB for intracellular transport and secretion [33,34], which would be inhibited by brefeldin.

### Memory CD4 T cells with granzyme B production capacity include Tregs and Th1 cells, with potentially more distinct GrzB vs. cytokine secretory mechanisms compared to CD8 T cells

We lastly examined memory CD4 T cell subsets that produce GrzB such as Treg, Th1, Th2, and Th17 cells. Memory CD4+CD45RO+ T cells were purified from peripheral blood and activated (CD3/CD28 costimulation or PMA/IO in the presence of brefeldin) or UT for 24 hrs. Cells were then analyzed by flow cytometry for phenotype (CD25-high and Foxp3 for Tregs) and function (intracellular cytokines - IFNγ for Th1, IL4 for Th2, and IL17A for Th17 cells) in conjunction with GrzB expression.

**Figure 5:**
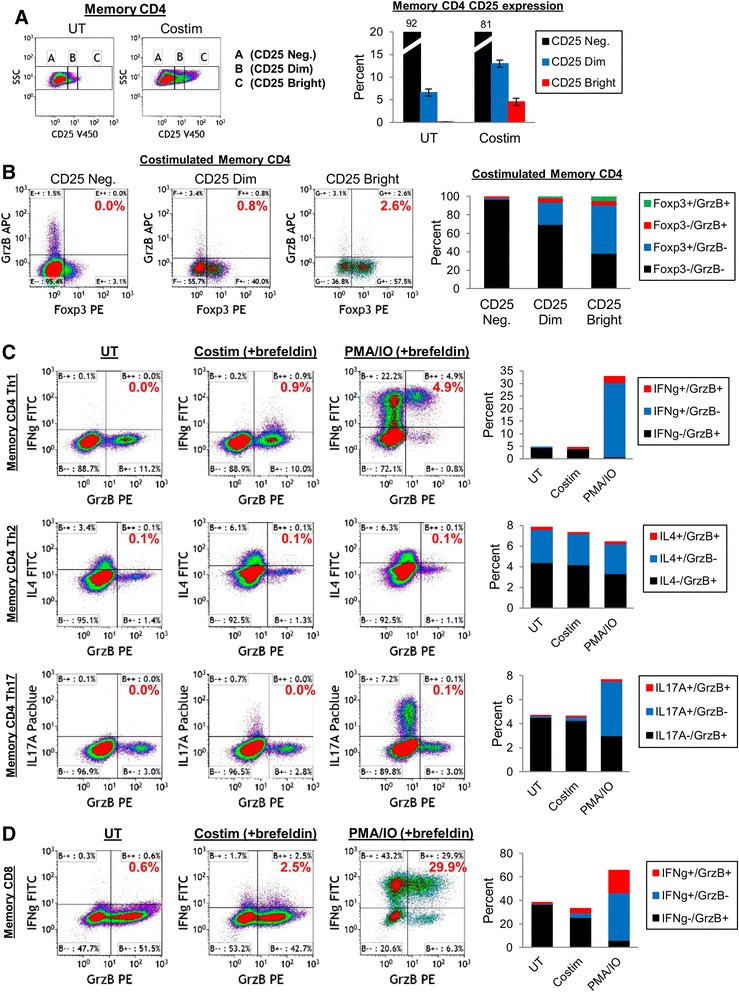
**GrzB production by memory CD4 T cell subsets and coexpression with cytokines. (A-B)** GrzB expression by Tregs. Memory CD4 T cells were purified from peripheral blood, and 5×10^5^ cells (in 1ml medium) were untreated (UT) or activated (CD3/CD28 costimulation) for 24 hrs. Cells were then stained for CD25, Foxp3, and GrzB. **(A)** CD25 expression levels (Negative, Dim, or Bright) by memory CD4 T cells after 24 hrs culture (mean ± sem, n = 3). **(B)** Foxp3/GrzB expression by CD25-Negative, CD25-Dim, or CD25-Bright memory CD4 T cells after 24 hrs culture. Graph shows the mean (n = 3) distribution of Foxp3/GrzB subsets gated on CD25-Negative, -Dim, or -Bright cells. **(C)** GrzB coexpression with IFNγ, IL4, or IL17A by memory CD4 T cells. Memory CD4 T cells (5×10^5^ cells in 1ml medium) were untreated for activated (CD3/CD28 costimulation or PMA/IO in the presence of brefeldin) for 24 hrs, then stained for GrzB and either IFNγ, IL4, or IL17A. Graphs show the mean (n = 3) distribution of cytokine/GrzB subsets. **(D)** GrzB/IFNγ coexpression by memory CD8 T cells. Purified memory CD8 T cells (5×10^5^ cells in 1 ml medium) were untreated or activated (CD3/CD28 costimulation or PMA/IO in the presence of brefeldin) for 24 hrs. Graph shows the mean (n = 3) distribution of IFNγ/GrzB subsets.

24 hrs costimulation or PMA/IO treatment of memory CD4 T cells resulted in GrzB expression by phenotypes consistent with Treg and Th1 cells (Figure 5A-C). Tregs were examined by measuring intracellular GrzB expression by CD25+/Foxp3+ costimulated memory CD4 T cells. Activated memory CD4 T cells expressed ~18% CD25 (13% CD25-Dim and 4.6% CD25-Bright, Figure 5A). GrzB was coexpressed with Foxp3 by CD25-Dim cells (24.2 ± 3.6% Foxp3+/GrzB-, 4.6 ± 1.0% Foxp3-/GrzB+, and 1.4 ± 0.4% Foxp3+/GrzB+), and more so by CD25-Bright cells (52.5 ± 4.0% Foxp3+/GrzB-, 4.8 ± 0.8% Foxp3-/GrzB+, and 4.3 ± 0.6% Foxp3+/GrzB+, Figure 5B). This is consistent with reports that demonstrate Treg expression of GrzB for suppressive functions [35-37]. IFNγ was modestly induced and coexpressed with GrzB by costimulated memory CD4 T cells (0.3 ± 0.1% IFNγ+/GrzB- and 0.5 ± 0.3% IFNγ+/GrzB+), but more robustly by PMA/IO treatment (29.6 ± 3.4% IFNγ+/GrzB- and 2.4 ± 0.8% IFNγ+/GrzB+, Figure 5C), indicating that memory CD4 Th1 cells express GrzB, consistent with other reports [38-41]. By contrast, IL4 was not induced by costimulated or PMA/IO-treated memory CD4 T cells (0.1% IL4+/GrzB+), suggesting that GrzB is not expressed by memory CD4 Th2 cells. IL17A was induced by costimulation and PMA/IO, but not coexpresssed with GrzB (0.1% IL17A+/GrzB+), suggesting that in these short-term conditions memory CD4 Th17 cells may not express GrzB. Th17 cells are reported to express GrzB in vivo [42,43], which likely involves more complex stimuli, timing or differentiation, and more specific phenotypic characterization (ie. CCR6, CD161, and IL-23R). Thus, Tregs and Th1 cells are subsets of peripheral blood memory CD4 T cells that produce GrzB in the present study, although other GrzB-producing subsets, particularly CD4 CTL’s, are likely involved as well [8-10,14].

For comparison of cytokine/GrzB coexpression by memory CD4 Th1 cells, purified memory CD8+CD45RO+ T cells were examined for IFNγ and GrzB coexpression as well (Figure 5D). Compared to CD8 T cells or NK cells, cytokine secretion pathways of CD4 T cells may be more diversified and distinct from those of cytotoxic mediators such as granzymes [44]. For example, in activated murine CD4 T cells, IL2 and IFNγ colocalize and are secreted by a more polarized mechanism compared to other secreted mediators such as TNFα and chemokines [45]. Additionally in NK cells, IFNγ and TNFα are stored and packaged in vesicles distinct from perforin-containing granules, and secreted in a more nonpolarized fashion compared to perforin [46]. In the present study, 24 hrs CD3/CD28 costimulation of memory CD8 T cells induced 3.8 ± 1.5% IFNγ+/GrzB- and 3.7 ± 2.0% IFNγ+/GrzB+ expression, and PMA/IO treatment induced 40.1 ± 5.3% IFNγ+/GrzB- and 19.3 ± 5.9% IFNγ+/GrzB+ expression. Thus, compared to PMA/IO-treated memory CD4 T cells (~3% IFNγ+/GrzB+), IFNγ+/GrzB+ expression by PMA/IO-treated memory CD8 T cells was significantly higher (p < 0.05), suggesting that cytokine, as well as perforin, secretion pathways of memory CD4 T cells may be more disassociated with GrzB secretion compared to memory CD8 T cells. These differences may be relevant to the weak correlations of GrzB expression and secretion between memory CD4 and memory CD8 T cells within single donors shown in Figure 2G-I.

## Conclusions

In the present study, GrzB expression and secretion was directly compared between human memory CD4 and memory CD8 T cells using flow cytometry, ELISpot, and ELISA. The results show that memory CD8 T cells store more GrzB, but do not secrete more GrzB, compared to memory CD4 T cells during short-term activation, suggesting that activated CD8 T cells regulate their GrzB release more than CD4 T cells. Consequently, the correlation between intracellular and extracellular GrzB for memory CD8 T cells is weaker compared to memory CD4 T cells. Additionally, CD107a is a better indicator of GrzB secretion by memory T cells than intracellular GrzB itself. These results corroborate and further clarify previous studies examining GrzB production by human or mouse CD4 and CD8 T cells, which utilized either flow cytometry, ELISpot, or ELISA, but not all three methods simultaneously. Distinct patterns of GrzB production between memory T cells may further be due to different associations with other secreted factors such as perforin or cytokines.

## Abbreviations

CTL: Cytotoxic T lymphocyte
LAMP-1: Lysosomal-associated membrane protein1
TCR: T cell receptor
